# The Russian invasion of Ukraine and its public health consequences

**DOI:** 10.1016/j.lanepe.2022.100358

**Published:** 2022-03-08

**Authors:** David A. Leon, Dmitri Jdanov, Christopher J. Gerry, Pavel Grigoriev, Domantas Jasilionis, Martin McKee, France Meslé, Olga Penina, Judyth Twigg, Jacques Vallin, Denny Vågerö

**Affiliations:** a Department of Non-communicable Diseases Epidemiology, London School of Hygiene and Tropical Medicine, UK; b Max Planck Institute for Demographic Research, Rostock, Germany; c University of Oxford, School of Global and Area Studies, UK; d Federal Institute for Population Research, Wiesbaden, Germany; e Max Planck Institute for Demographic Research, Rostock, Germany; f Demographic Research Centre, Vytautas Magnus University, Kaunas, Lithuania; g Department of Health Services Research and Policy, London School of Hygiene and Tropical Medicine, UK; h French Institute for Demographic Studies (INED), Paris, France; i Nicolae Testemitanu State University of Medicine and Pharmacy, Republic of Moldova; j Department of Political Science, Virginia Commonwealth University, Richmond, USA; k French Institute for Demographic Studies (INED), Paris, France; l Department of Public Health Sciences, Stockholm University, Sweden

The unprovoked and unjustified Russian invasion of Ukraine on 24 February 2022 is already having terrible consequences for health. As we write, only seven days since Russian troops crossed the border, substantial numbers on both sides have died, many due to Russian attacks on civilian targets in violation of international law.^1^

This catastrophic turn of events comes hard on the heels of the two-year COVID-19 pandemic during which countries of Eastern Europe were among some of the worst affected.^2^ All governments are struggling to replenish public finances, with health services facing great pressures, yet the invasion is resulting in substantial resources in Europe now being understandably diverted to bolster defences.^3^

The Ukrainian health system is facing exceptional pressure. Demand has vastly increased by the steeply growing number of casualties caused by the attacks on densely populated areas, alongside the ongoing needs of those with serious infectious and chronic conditions. However, its ability to meet these needs is being severely depleted by the reckless destruction of infrastructure, displacement of health workers and shortage of essential supplies, including oxygen.

The physical and mental health problems experienced by the anticipated millions of refugees and internally displaced persons will once again be substantial.^4^ This is a major challenge that European countries appear to be responding to, although it is a sad reality that those most in need will be the least likely to make the arduous and sometimes dangerous journey to find safety outside of Ukraine. The long-term scars of physical injury and psychological trauma suffered by millions in Ukraine will be felt long after this war is past.

The impact of these events will also be felt in the months to come by the people of Russia and Belarus following imposition of unprecedented economic sanctions. Their health systems will face severe shortages, including modern therapies, many of which come from Western countries. Beyond this, the social, economic and political stresses will worsen the living conditions of many, and may potentially trigger substantially increased mortality similar to that seen following the collapse of the Soviet Union.

We have spent decades collaborating with colleagues in Eastern Europe and the former Soviet Union to understand why population health in these countries has historically lagged behind the West and what can be done to close the gap. This has been motivated by a desire to improve the health and welfare of ordinary people and not to protect, burnish or advance the reputations of governments or political leaders.

Along with much of the world we call for an end to the fighting and for Russian forces to withdraw immediately before causing further death and injury. However, the invasion of Ukraine raises particular questions for all scientists who have worked collaboratively in the region. We need to support, morally and practically, our Ukrainian colleagues who face threats to their lives. We welcome the decision of the European Union to offer sanctuary to all Ukrainians. For Ukrainian researchers and academics who leave their country we also need a special international programme that provides them with a supportive professional environment.

We note how several thousand scientists in Russia have signed a letter opposing the invasion, despite the risks this entails to their careers and possibly their liberty.^6^ Those who are in danger due to their political views or speak out against the war should be supported by their international colleagues. More generally, we believe that contacts between individual researchers should be maintained and work continued to the extent that it is possible, and support the decision by Elsevier that its journals should not simply reject a scientific paper because it is from Russian researchers.^5^ However, for now, institutional links between universities and research institutes in Russia and other countries, enshrined in formal agreements, should be suspended.

Finally, this war is not just about Ukraine and Russia. The threat of Russian expansion, with massive consequences for global security and sustainable development, coincides with other crises facing humanity including climate change and the prospect of future pandemics. The world should be coming together to tackle these existential global issues rather than being forced to respond to this disastrous war and the humanitarian crisis it is already creating.

## Author contribution

The initial text was written by DAL and DJ with crucial input from other colleagues. Extensive suggestions and changes were made by all other authors over the course of a series of revisions all of whom contributed equally. All authors approved the final version prior to submission.

## Declaration of interests

Authors do not have anything to declare.
